# Limitations of Thermal Stability Analysis via *In-Situ* TEM/Heating Experiments

**DOI:** 10.3390/nano11102541

**Published:** 2021-09-28

**Authors:** Osman El-Atwani, Hyosim Kim, Cayla Harvey, Mert Efe, Stuart A. Maloy

**Affiliations:** 1 Materials Science and Technology, Los Alamos National Lab, Los Alamos, NM 87545, USA; hkim@lanl.gov (H.K.); cayla@lanl.gov (C.H.); maloy@lanl.gov (S.A.M.); 2 Chemical and Materials Engineering, University of Nevada, Reno, NV 89557, USA; 3 Energy and Environment Directorate, Pacific Northwest National Laboratory, Richland, WA 99354, USA; Mert.efe@pnnl.gov

**Keywords:** nanocrystalline, ultrafine, thermal stability, *in-situ* TEM, stagnation

## Abstract

This work highlights some limitations of thermal stability analysis via *in-situ* transmission electron microscopy (TEM)-annealing experiments on ultrafine and nanocrystalline materials. We provide two examples, one on nanocrystalline pure copper and one on nanocrystalline HT-9 steel, where *in-situ* TEM-annealing experiments are compared to bulk material annealing experiments. The *in-situ* TEM and bulk annealing experiments demonstrated different results on pure copper but similar output in the HT-9 steel. The work entails discussion of the results based on literature theoretical concepts, and expound on the inevitability of comparing *in-situ* TEM annealing experimental results to bulk annealing when used for material thermal stability assessment.

## 1. Introduction

Microstructural stability assessment of materials exposed to thermal loads is an essential criterion in climbing the technology readiness level and qualifying materials for applications in industry. Possible microstructural instabilities (e.g., grain size growth or recrystallization) can alter the mechanical properties of materials [1,2]. For example, the hardness, toughness and ductility of materials are grain size dependent [3] and understanding the dependency of these properties as a function of grain size is a crucial step to design thermally stable materials for different applications. A class of materials which has demonstrated to offer significant advantages over commercial materials in terms of mechanical properties and radiation resistance (for nuclear industry) is nanocrystalline (NC), with grain sizes below 100 nm, and ultrafine (UF), with grain sizes in the range of 100–500 nm, grained materials [4,5]. NC and UF grained metallic materials are discussed as possible fusion and fission reactor materials, which have different material requirements and operating conditions [6]. However, these materials have to possess thermal stability and avoid recrystallization. For example, in the Demonstration (DEMO) power plant for fusion, the surface temperature of the divertor is designed to be less than the recrystallization temperature of tungsten (a primary candidate as a plasma facing material) [7,8]. Tungsten in the recrystallized conditions is not favorable due to its low strength, low thermal shock resistance and high ductile to brittle transition temperature (DBTT) [9].

As a rapid characterization technique to assess thermal stability of NC and UF grained materials, performing *in-situ* transmission electron microscopy (TEM)/heating experiments has evolved, where thin specimen (preferably below 100 nm) are heated inside the TEM microscope while observing the changes in morphology. These experiments were used on different materials and the thermal stability of these materials in the pristine conditions and irradiated conditions (for nuclear materials) are concluded in some cases based on the *in-situ* TEM/heating observations [10,11,12,13,14,15].

In this work, we study two material systems (NC copper, polycrystalline Cu, and NC HT-9 steel) under *in-situ* TEM/heating and bulk heating (with *ex-situ* characterization of morphology) and demonstrate differences on how *in-situ* TEM/heating experiments can show a limited picture of NC materials behavior under thermal loads. The work on these selected materials of some distinct microstructures has allowed one to discuss several mechanisms suggested in literature regarding the stability of NC and UF grained materials in their bulk and thin film forms and clearly elucidate some limitations of *in-situ* TEM/heating experiments. The goal of this paper is not to reveal the grain growth kinetics in these NC materials, but rather to increase awareness of the limitations of *in-situ* TEM experiments, discussed from literature concepts, and to elucidate how data analysis of *in-situ* of this type of experiments should always be compared to *ex-situ* bulk heating results in some cases.

## 2. Materials and Methods

*In-situ* TEM/heating and bulk heating (under vacuum furnace) experiments were performed on pure NC Cu and HT-9 steels. The main elements in the HT-9 steel, which is a ferritic/martensitic steel, were Cr ~12%. 0.5% W, 0.3% V, 1% Mo, 0.55% Ni, 0.25% Si, 0.55% Mn, 0.2% C and the balance was Fe. The NC materials were prepared via large strain machining (LSM) [16] at RT with a zero rake angle and a speed of 115 RPM. The LSM samples were first cut into 3 mm discs and then mechanically polished to ~100 µm thickness. TEM samples of the NC Cu and NC HT-9 were then prepared via electropolishing, the mechanically polished discs, with 10% phosphoric acid/water (at RT) and 5% perchloric acid/methanol (at −30 °C) solutions respectively. For bulk heating, the mechanically polished discs were electropolished for ~10 s to eliminate any possible damage from mechanical polishing. The *in-situ* TEM heating experiments were performed using a built-in furnace holder and a ramp rate of 23.3 degrees/min to 700 °C (or ramping time of 30 min). Bulk heating experiments were performed inside a vacuum chamber with a similar ramping rate. Investigation of morphologies before and after heating was performed using TEM and electron backscattered diffraction (EBSD). Morphology characterization was performed on thin (<100 nm thickness) and thick (10 s of µms thickness) regions of the TEM samples and on the bulk heated samples.

## 3. Results

**Figure 1:**
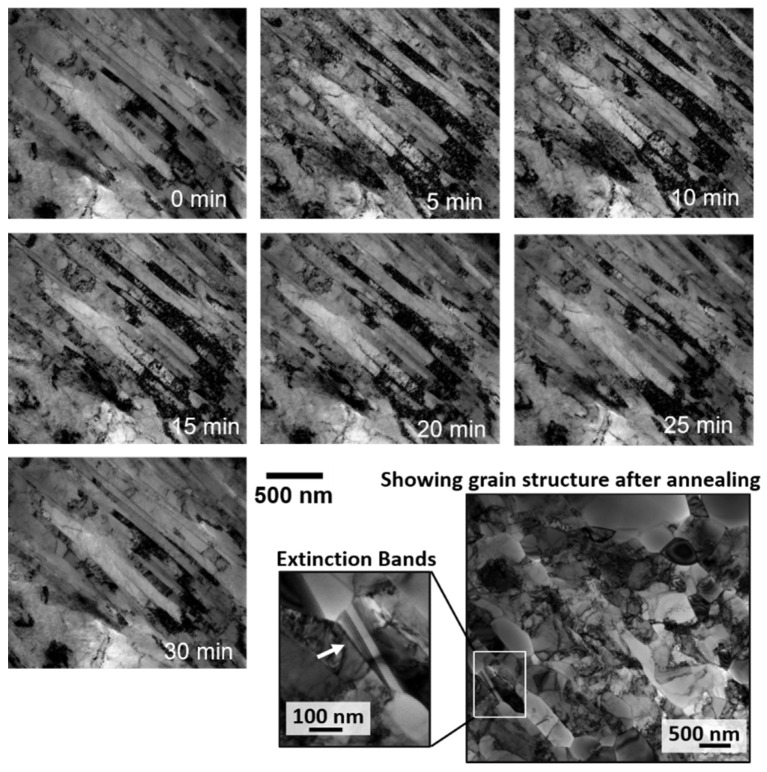
TEM micrographs of *in-situ* annealing of NC Cu from 0 to 700 °C in 30 min. The bottom right image shows a lower magnification image of a grain structure (after annealing) in a different thin area of the sample with some non-equilibrium grains marked by extinction bands.

**Figure 2:**
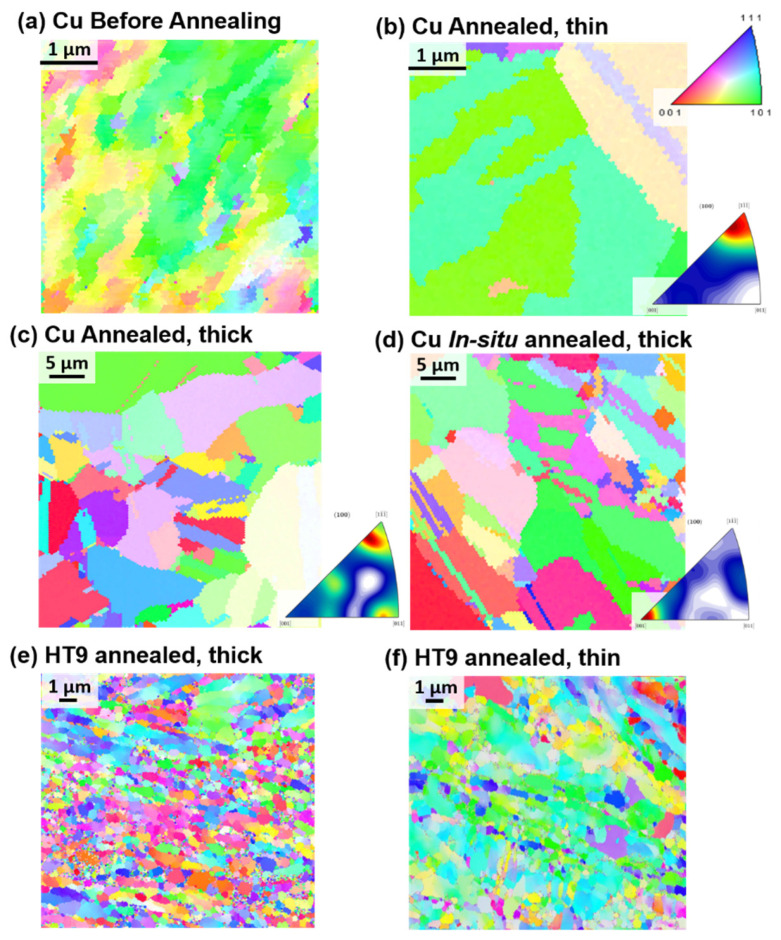
Inverse pole figure EBSD maps for (**a**–**c**) LSM Cu before and after annealing in thick and thin regions. (**d**) LSM bulk Cu material annealed. (**e**,**f**) HT-9 annealed in thick and thin regions.

**Figure 3:**
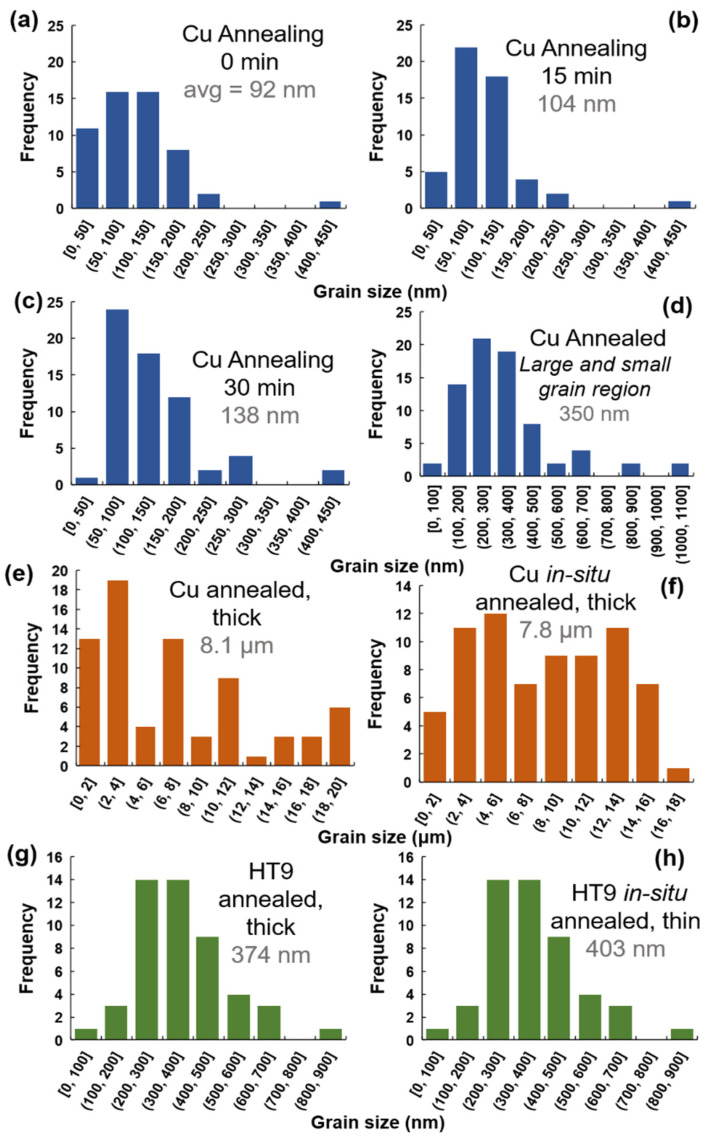
Grain size histograms for (**a**–**d**) *in-situ* Cu annealing as shown in Figure 2, with (**d**) showing grain size after annealing in a larger area of the thin area in the sample. (**e**,**f**) show Cu annealed corresponding to the EBSD (**c**,**d**) in Figure 2 respectively. (**g**,**h**) HT9 annealed corresponding to (**e**,**f**) in Figure 2 respectively.

Bright-field micrographs from the *in-situ* TEM experiments on the NC Cu sample as a function of time are shown in Figure 1. EBSD results are shown in Figure 2. The corresponding change in grain size (calculated from TEM bright-field images or EBSD results) during the course of the experiment is shown in Figure 3. It was evident that the sample microstructures, governed by elongated NC and UF grains, were very thermally stable with a small grain size increase starting to occur at 450 °C (Figure 1 and Figure 3). Under *ex-situ* heating at 400 °C and for a similar ramping and annealing time, the NC Cu sample showed large grain growth as demonstrated from the EBSD results in Figure 2c (corresponding change in grain size is plotted in Figure 3e). Further examination of the thin and thick regions in the *in-situ* TEM annealed sample demonstrated grain growth in some parts of the thin region (while other grains showed no grain growth) (Figure 1 and Figure 2b) while the thick regions showed large grain growth (Figure 2d). The corresponding changes in the grain size are plotted in Figure 3d,f respectively.

Similar experiments were performed on NC HT-9 as shown in Figure 2. While some grains showed no grain growth, other grains in the thin area demonstrated moderate grain size increase (Figure 2f and Figure 3h) but similar results and grain sizes to the *ex-situ* heating (Figure 2e and Figure 3g). Details of the microstructure of the pristine sample (prior to annealing) are shown in ref. [17].

## 4. Discussion

In the *in-situ* NC Cu case, where elongated grains showed no grain growth, grain size stagnation occurred. This phenomenon was studied and illustrated in literature [18]. While normal grain growth, or curvature driven grain growth [19,20] is associated with reduction of grain boundary energy, which is dependent on the macroscopic degrees of freedom of the grain boundary, the presence of surfaces in thin films or thin specimens introduces other driving forces for grain growth such as the reduction of surfaces and elastic strain energies [18]. Samples formed through deformation, as is the case in this work, have also stored energy due to deformation (related to grain size distribution) and residual stresses that can induce grain growth. The retarding forces to grain growth driving forces are impurities [21] and grain boundary grooving [22]. In the pure NC Cu, it is expected that grain boundary grooving, formed though the balance of surface and grain boundary forces, is causing grain size stagnation [23] Grain boundary stagnation is expected when the grain size to the film thickness is within the ratio of 1–3 [24] which is the case in the ~100 nm thickness samples used during TEM experiments.

Frost [24] discussed a critical curvature for the grain boundary to pass or climb a groove as:

(1) $$K_{crit}=\frac{\gamma_{gb}}{\gamma_{s}h}$$

where $\gamma_{gb}$ and $\gamma_{s}$ are grain boundary and surface energies respectively and *h* is the film thickness. It is evident from the equation that decreasing the film thickness is associated with an increase in the critical curvature. As shown in Figure 1 and Figure 2, the elongated grains which showed no grain growth during the experiments are textured and the grain boundaries are of low angle and hence, of low energy which should be associated with lowering the critical curvature. Moreover, these elongated grain boundaries are expected to have low grain boundary velocity, which is proportional to *1*/*r* [25] where r is the radius of curvature, due to the large curvature of the elongated boundaries. Still under *ex-situ* irradiation, these grain boundaries were not stagnant and evolved into equiaxed large grain boundaries due to the secondary growth [26]. Severe plastically deformed materials are usually expected to possess non-equilibrium boundaries, which are high angle boundaries associated with high density of extrinsic dislocations [27]. While the non-equilibrium state can provide high atomic mobility and lead to lower activation energy for grain boundary migration [28], the recovery of these non-equilibrium grain boundaries to equilibrium counter parts during annealing can lead to an increase in the activation energy and a significant decrease in the driving force and grain boundary mobility, and thus an increase in the grain boundary thermal stability [29]. Based on the EBSD results from the NC Cu prior to annealing (Figure 2), the NC Cu is formed mainly of elongated low angle boundaries while non-equilibrium boundaries, marked by extinction bands formed after grain growth and were associated with the largest grains after the experiments (Figure 1). Therefore, the stagnation of the grain boundaries in the NC Cu is independent of the effect of high angle non-equilibrium boundaries.

After 600 °C in the *in-situ* experiments, grain growth occurred and at 700 °C (Figure 1 and Figure 2b), large grains were observed adjacent to thermally stable and small grains. This is a result of the secondary recrystallization [26], in which the material’s surfaces play a dominant role during grain growth where the thickness is comparable to the grain size. [18]. Some grains with preferable orientation grow rapidly to minimize their surface energy during growth [18,30]. This leads to bimodal distribution of grains due to the energy anisotropy [18,26] as shown in Figure 3. Images of rapidly grown grains near stagnant grains are shown in Figure 1 and Figure 2. The secondary grain growth is also associated with the development of the sample texture [31,32,33] due to the selective rapid grain growth of certain orientations as mentioned earlier. This is also demonstrated in Figure 2b for the thin area heated *in-situ* when compared to the *ex-situ* bulk material results (Figure 2c) or the thick region of the *in-situ* results (Figure 2d). This is due to the large thickness of the thick area or the *ex-situ* bulk heated sample where grains are expected to follow normal grain growth.

In the case of the NC HT-9, the morphology of the sample consisted of elongated and equiaxed grains. The *in-situ* TEM annealing was performed on the equiaxed grains while also observing the elongated grains intermittently. From the *in-situ* data [17], some grain growth and recrystallization occurred where the recrystallized grains demonstrated further thermal stability. Both the *in-situ* and the *ex-situ* annealing lead to similar grain size (Figure 2e,f and Figure 3g,h) and no secondary growth or texture development occurred in the *in-situ* annealed experiments. This proves the stability of these films in which *in-situ* TEM truly predicted the material’s thermal stability. Stability of ultrafine grains with impurities are attributed to the impurity drag effect (Zener pressure) [34] which stabilizes the grain and opposes the grain growth driving force [26]. The EDX of the NC HT-9 has demonstrated carbide formation on the grain boundaries and the thermal stability of these grains were attributed to lower dislocation densities and presence of carbides [17]. In a NC pure material case, El-Atwani et al. has shown stable ~100–200 nm grain size NC pure Fe grains up to temperature 600 °C [35]. These results were consistent with other works on bulk NC Fe [36,37] demonstrating another case where *in-situ* TEM predicted the bulk material behavior. However, it is evident that NC materials that are stable in bulk forms are also stable during *in-situ* TEM experiments, but the opposite may not be true.

## 5. Conclusions

The two examples provided in this work and the corresponding comparison and illustration of established theories in literature provide sufficient evidence that vigilant analysis and comparison with bulk heating experiments should be performed to establish kinetics and conclusions regarding thermal stability of NC and UF materials via *in-situ* TEM/heating experiments, which have to be performed on thin areas that are usually below 100 nm thicknesses. Grain size stagnation and secondary grain growth which can occur in NC and UF materials have to be investigated and if shown to exist, then subsequent conclusions can only be drawn in regards to the thin film form of the investigated material.

## Data Availability

The data presented in this study are available within the article itself.
